# MiR-454-3p Promotes Oxaliplatin Resistance by Targeting PTEN in Colorectal Cancer

**DOI:** 10.3389/fonc.2021.638537

**Published:** 2021-05-04

**Authors:** Xiao-Lan Qian, Fang Zhou, Song Xu, Jian Jiang, Zhi-Peng Chen, Shao-Kai Wang, Yun Zuo, Chen Ni

**Affiliations:** Department of Oncology, Zhangjiagang First People's Hospital, The Affiliated Zhangjiagang Hospital of Soochow University, Zhangjiagang, China

**Keywords:** colorectal cancer, oxaliplatin resistance, miR-454-3P, PTEN, AKT signaling pathway

## Abstract

Colorectal cancer is one of the most common malignancies worldwide. Oxaliplatin is the first-line chemotherapeutic agent for the treatment of advanced colorectal cancer. However, acquired resistance to oxaliplatin limits its therapeutic efficacy, and the underlying mechanism remains largely unclear. In this study, we compared the expression of a panel of microRNAs (miRNAs) between oxaliplatin-sensitive and -resistant HCT-116 colorectal cancer cells. We found that miR-454-3p was significantly up-regulated in oxaliplatin-resistant cells and was the most differently expressed miRNA. Interestingly, we observed that inhibition of miR-454-3p resensitized resistant cells to oxaliplatin and enhanced oxaliplatin-induced cellular apoptosis. Moreover, we determined that miR-454-3p promoted oxaliplatin resistance through targeting PTEN and activating the AKT signaling pathway. *In vivo* study revealed that overexpression of miR-454-3p decreased the sensitivity of HCT-116 xenograft tumors to oxaliplatin treatment in a mouse model. Clinically, overexpression of miR-454-3p was associated with decreased responsiveness to oxaliplatin-based chemotherapy, as well as a short progression-free survival. Taken together, our study indicated that the expression of miR-454-3p could be used to predict oxaliplatin sensitivity, and targeting miR-454-3p could overcome oxaliplatin resistance in colorectal cancer.

## Introduction

Colorectal cancer is the third most commonly diagnosed cancer and the second leading cause of cancer death, accounting for ~10% of all cancer cases and deaths worldwide (1). Although surgery is considered the only curative treatment for colorectal cancer, chemotherapy remains the most widely used monotherapy or adjuvant therapy for the majority of patients (2, 3). Oxaliplatin, a platinum-based derivative, is the first-line chemotherapeutic agent for the treatment of advanced colorectal cancer (4, 5). However, drug resistance often occurs after a short period of use of oxaliplatin, which leads to therapeutic failure and poor prognoses. Drug resistance limits the efficacy of oxaliplatin and remains a major obstacle to the effective treatment of colorectal cancer (6, 7). Therefore, it is urgent to investigate the underlying molecular mechanism for the development of novel therapeutic strategy to overcome oxaliplatin resistance in colorectal cancer.

MicroRNAs (miRNAs) are a class of small non-coding RNAs consisting of 17–25 nucleotides. MiRNAs regulate gene expression by directly binding to the 3′-untranslated region (UTR) of target messenger RNAs (mRNAs), triggering these mRNAs to degrade or inhibiting their translation (8). In recent years, increasing studies have confirmed the critical roles of aberrantly expressed miRNAs in chemotherapeutic drug resistance, including oxaliplatin (9–12). However, the molecular mechanism underlying the resistance of colorectal cancer to oxaliplatin modulated by miRNAs was not clearly understood.

In the present study, we identified that miR-454-3p was significantly up-regulated in oxaliplatin-resistant colorectal cancer cells, as compared to that in oxaliplatin-sensitive cells. Inhibition of miR-454-3p increased the antiproliferative activity of oxaliplatin and enhanced cellular apoptosis in oxaliplatin-resistant cells both *in vitro* and *in vivo*. Furthermore, we showed that miR-454-3p promoted oxaliplatin resistance by directly targeting PTEN, thereby down-regulating its expression and activating the AKT signaling pathway. More importantly, we demonstrated that high levels of miR-454-3p expression were negatively correlated with the clinical response to oxaliplatin-based chemotherapy, as well as progression-free survival (PFS). These findings will enhance our understanding of the molecular mechanism underlying the chemoresistance of colorectal cancer and support that miR-454-3p may possess the potential of predicting oxaliplatin sensitivity in colorectal cancer.

## Materials and Methods

### Cell Culture and Reagents

The human colorectal cancer cell line HCT-116 was obtained from the Cell Bank of the Chinese Academy of Sciences (Shanghai, China). Cells were cultured in RPMI-1640 medium (Thermo Scientific, Rockford, IL, USA) supplemented with 10% fetal bovine serum (Thermo Scientific) and 1% penicillin–streptomycin (Thermo Scientific) at 37°C in a humidified incubator with 5% CO_2_. Oxaliplatin-resistant colorectal cancer cell line HCT-116/OxR was established in our laboratory through dosage escalation of oxaliplatin beginning from 100 nM. Thereafter, the concentrations of oxaliplatin were elevated in gradient until the cells could stably proliferate in 10 μM of oxaliplatin. HCT-116/OxR cells were cultured in the medium containing 5 μM of oxaliplatin. Oxaliplatin and LY294002 were purchased from Sigma (St. Louis, MO, USA), dissolved in phosphate-buffered saline (PBS) or dimethyl sulfoxide (DMSO), and stored at 4°C or −20°C, respectively.

### Cell Transfection

The miR-454-3p mimic (5′-UAGUGCAAUAUUGCUUAUAGGGU-3′), negative control mimic (miR-NC, sequence: 5′-UCACAACCUCCUAGAAAGAGUAGA-3), miR-454-3p inhibitor (anti–miR-454-3p inhibitor, sequence: 5′-ACCCUAUAAGCAAUAUUGCACUA-3′), negative control inhibitor (anti–miR-NC, sequence: 5′-CAGUACUUUUGUGUAGUACAA-3′), and small interfering RNA (siRNA) for PTEN, as well as corresponding negative control (siNC) were obtained from GenePharma (Shanghai, China). For PTEN overexpression, PTEN cDNA sequence was cloned into pcDNA 3.1 vector plasmid. All transfection experiments were performed using Lipofectamine 2000 (Invitrogen, Carlsbad, CA, USA) according to manufacturer's protocol. For stable miR-454-3p overexpression cells, lentiviral plasmids encoding miR-454-3p and negative control (miR-NC) were transfected into 293T packaging cells to generate lentiviruses. HCT-116 cells were infected by the lentiviruses for 24 h and were then selected with 2 μg/mL puromycin for at least 3 days.

### Real-Time Polymerase Chain Reaction

Total RNA was extracted from cells using miRNeasy mini kit (Qiagen, Hilden, Germany) and was reversely transcribed to complementary DNA using TaqMan miRNA reverse transcription kit (Applied Biosystems, Foster City, CA, USA) according to the manufacturer's instructions. Quantitative real-time polymerase chain reaction (qRT-PCR) was performed using TaqMan miRNA Assays (Applied Biosystems). *U6* was used as an endogenous control, and the expression of miR-454-3p was quantified using the 2^−ΔΔCt^ method. The real-time PCR primers of miR-454-3p were used as follows: forward: 5′-GCGCGTAGTGCAATATTGCTTA-3, reverse: 5′-AGTGCAGGGTCCGAGGTATT-3, *U6* forward: 5′-TGCGGTGGGTGTCATCAAA-3′, and reverse: 5′-AACGCTTCACGAATTTGCGT-3′.

### Cell Viability Assay

After transfection of miR-454-3p mimics, inhibitor, or corresponding controls, cells were seeded in 96-well plates at a density of 4 × 10^3^ cells per well and incubated at 37°C overnight. The cells were then treated with indicated concentrations of oxaliplatin or PBS (negative control) for 48 h. Then, 20 μL medium containing MTT (5 mg/mL; Sigma) was added to each well, and the cells were incubated for another 4 h at 37°C. The MTT containing medium was discharged, and 150 μL DMSO was added to each well to dissolve the new formed formazan. Light absorbance at 570 nm was measured on a microplate reader (Synergy H4, BioTek). The results were represented as mean ± SD from three independent experiments.

### Cell Apoptosis

After transfection, cells were treated with indicated concentrations of oxaliplatin or LY294002 for 48 h. For cellular apoptosis qualification, the cells were harvested and stained with annexin V–fluorescein isothiocyanate (FITC) and propidium iodide (PI) for 15 min in the dark. Then, the stained cells were analyzed by flow cytometry to qualify cell apoptosis based on the percentage of annexin V–positive cells. For caspase-3 activity determination, caspase-3 colorimetric assay kits (Beyotime, Shanghai, China) were used according to the manufacturer's protocol. Briefly, cells were washed with cold PBS, resuspended in lysis buffer, and incubated on ice for 15 min. A total of 50 μL cell suspension, 40 μL reaction buffer, and 10 μL Ac-DEVD-pNA were mixed and then incubated at 37°C for 2 h. Light absorbance was measured at 405 nm. The Bradford protein quantitative analysis was used as the reference to normalize expression in each experimental group. The results were represented as mean ± SD from three independent experiments. Representative images from three independent experiments were shown.

### Western Blot

Cells were collected and lysed with RIPA lysis buffer. The concentrations of total protein were determined by BCA protein assay kit (Beyotime). Equal amounts of protein samples were subjected to sodium dodecyl sulfate–polyacrylamide gel electrophoresis for separation and were then transferred onto polyvinylidene difluoride membranes (Millipore, Billerica, MA, USA). The membranes were blocked with 1% skim milk for 1 h at room temperature, followed by incubation with primary antibodies at 4°C overnight. After incubation with corresponding secondary antibodies at room temperature for 1 h, the protein bands were detected with chemiluminescence (Millipore) according to manufacturer's instructions. β-Actin was used as an endogenous loading control. Representative data from at least three independent experiments were shown. Antibodies to PTEN, AKT, Bcl-2, Bax, and β-actin were purchased from Proteintech (Rosemont, IL, USA), Antibodies to p-AKT and cleaved caspase-3 were purchased from Cell Signaling Technology (Beverly, MA, USA). Representative images from three independent experiments were shown.

### Colony Formation Assay

After transfection, cells were seeded in a six-well plate at a density of 1 × 10^3^ cells per well and incubated at 37°C overnight. The cells were then treated with indicated concentrations of oxaliplatin or PBS (negative control) for 48 h, respectively. Then, fresh medium was replaced to allow cell growth for at least 2 weeks. After staining with gentian violet, colonies formed by more than 50 cells were counted. The results were represented as mean ± SD from three independent experiments.

### Luciferase Reporter Assay

The wild-type and mutant 3′-UTRs of PTEN were cloned into a PGL-3 control vector (Promega, Madison, WI, USA). Cells were seeded in 24-well plates and were cotransfected with firefly luciferase reporter vector containing wild-type or mutant 3′-UTRs of PTEN, along with miR-454-3p mimics or miR-NC in combination with pRL-SV40 Renilla luciferase vector using Lipofectamine 2000 (Invitrogen). After incubation at 37°C for 48 h, cells were harvested and analyzed for luciferase activity using dual-luciferase reporter assay system (Promega) according to the manufacturer's protocol. The signal of Renilla luciferase was used as an internal control to normalize the transfection efficacy. The results were represented as mean ± SD from three independent experiments.

### *In vivo* Tumor Xenograft Experiments

All animal experimental protocols were approved by the Medical Ethics Committee of Zhangjiagang First People's Hospital. Male BALB/c nude mice (6 weeks old, weight 18 ± 2 g) were purchased from Shanghai SLAC Laboratory Animal Co., Ltd., and were housed in a specific pathogen-free environment. The mice were randomly divided into two groups (*n* = 6) and subcutaneously injected with HC-116 cells (5 × 10^6^ in 200 μL PBS) stably transfected with miR-454-3p overexpression/negative control vector. The tumor volume was measured using a digital caliper and calculated as *L* × *S*^2^ × 0.52 (*L* represents the longest tumor diameter, and *S* represents the shortest tumor diameter). The tumor volume and mouse body weight were recorded every 2 days. After the tumors reached a mean volume of 100 mm^3^ (8 days after tumor inoculation), the animals began receiving oxaliplatin (5 mg/kg, intraperitoneally) every 4 days. At the end of experiment, the mice were sacrificed by cervical dislocation, and the tumors were harvested and weighed.

### Patients and Tumor Tissues

The study fusing human tissues was approved by the Medical Ethics Committee of Zhangjiagang First People's Hospital. A total of 45 human colorectal cancer samples were obtained from the Zhangjiagang First People's Hospital with the informed consent of each patient. All the patients were diagnosed with colorectal cancer based on pathological evidence and received at least six cycles of oxaliplatin-based chemotherapy. They were classified according to the Response Evaluation Criteria in Solid Tumors criteria. Clinicopathological information of the patients is presented in Table 1. All tissue samples for RNA extraction were snap-frozen and stored in liquid nitrogen. Levels of miR-454-3p expression in all tissues were normalized to the lowest level of expression. Subsequently, the median miR-454-3p expression in the colorectal cancer tissues was used as the cutoff value to divide the patients into two groups with high or low expression of miR-454-3p.

**Table 1:** Associations between miR-454-3p levels and clinicopathological characteristics.

**Variables**	**Expression of miR-454-3p**		***P*-value^a^**
	**High**	**Low**	
**Age (years)**			
<60	10	11	0.720
≥60	13	11	
**Gender**			
Male	11	10	0.855
Female	12	9	
**Tumor location**			
Colon	13	12	0.261
Rectum	10	11	
**Tumor size (cm)**			
<4	12	14	0.705
≥4	11	8	
**TNM stage**			
I/II	8	9	0.457
III/IV	15	13	
**Distant metastasis**			
No	17	18	0.691
Yes	6	4	
**Oxaliplatin response**			
Response	8	17	0.017^b^
Non-response	15	5	

a *P-value was estimated by χ^2^ test*.

b *Statistically significant*.

## Results

### The Expression of miR-454-3p Was Up-Regulated in Oxaliplatin-Resistant Colorectal Cancer Cells

**Figure 1:**
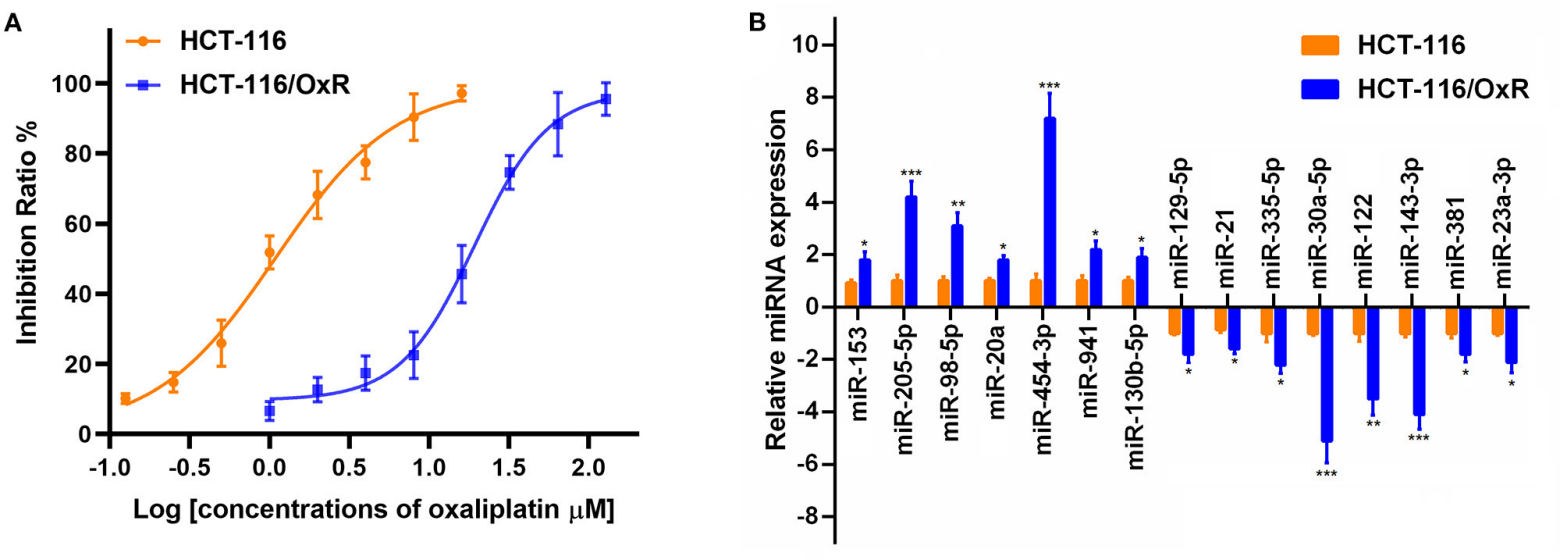
The expression of miRNAs in oxaliplatin-sensitive and -resistant HCT-116 colorectal cancer cells. **(A)** Effects of oxaliplatin on cell viability analyzed using MTT assay in HCT-116 and HCT-116/OxR cells. **(B)** Quantitative RT-PCR analysis on the expression of a panel of miRNAs between HCT-116 and HCT-116/OxR cells. *U6* small nuclear RNA was used as an internal control. Results were presented as mean ± SD from three independent experiments. *P < 0.05, **P < 0.01, ***P < 0.001.

To investigate the molecular mechanism underlying the oxaliplatin resistance in colorectal cancer cells, oxaliplatin-resistant HCT-116 cells (HCT-116/OxR) were established by culturing the HCT-116 cells with dosage escalation of oxaliplatin over 6 months. The effects of oxaliplatin on cell viability were analyzed using an MTT assay in HCT-116 and HCT-116/OxR cells. As shown in Figure 1A, HCT-116/OxR cells exhibited significant resistance to oxaliplatin, with an IC_50_ value ~20-fold larger than those of sensitive parental HCT-116 cells. To screen critical miRNAs associated with oxaliplatin resistance in colorectal cancer, we used quantitative RT-PCR assay to detect the expression of a panel of miRNAs composed of the most altered miRNAs in an miRNA microarray analysis (data not shown). As presented in Figure 1B, we found that seven miRNAs were up-regulated by more than 2-fold, whereas eight miRNAs were down-regulated by more than 2-fold in oxaliplatin-resistant cells, compared to oxaliplatin-sensitive cells. Specifically, the up-regulated miRNAs included miR-153, miR-205-5p, miR-98-5p, miR-20a, miR-454-3p, miR-941, and miR-130b-5p, whereas the down-regulated miRNAs included miR-129-5p, miR-21, miR-335-5p, miR-30a-5p, miR-122, miR-143-3p, miR-381, and miR-23a-3p. In particular, miR-454-3p was the most differentially expressed miRNA detected, with ~7-fold higher expression in HCT-116/OxR cells, compared with their parental cells. Therefore, miR-454-3p was selected for further studies.

### MiR-454-3p Induced Oxaliplatin Resistance in HCT-116 Colorectal Cancer Cells

**Figure 2:**
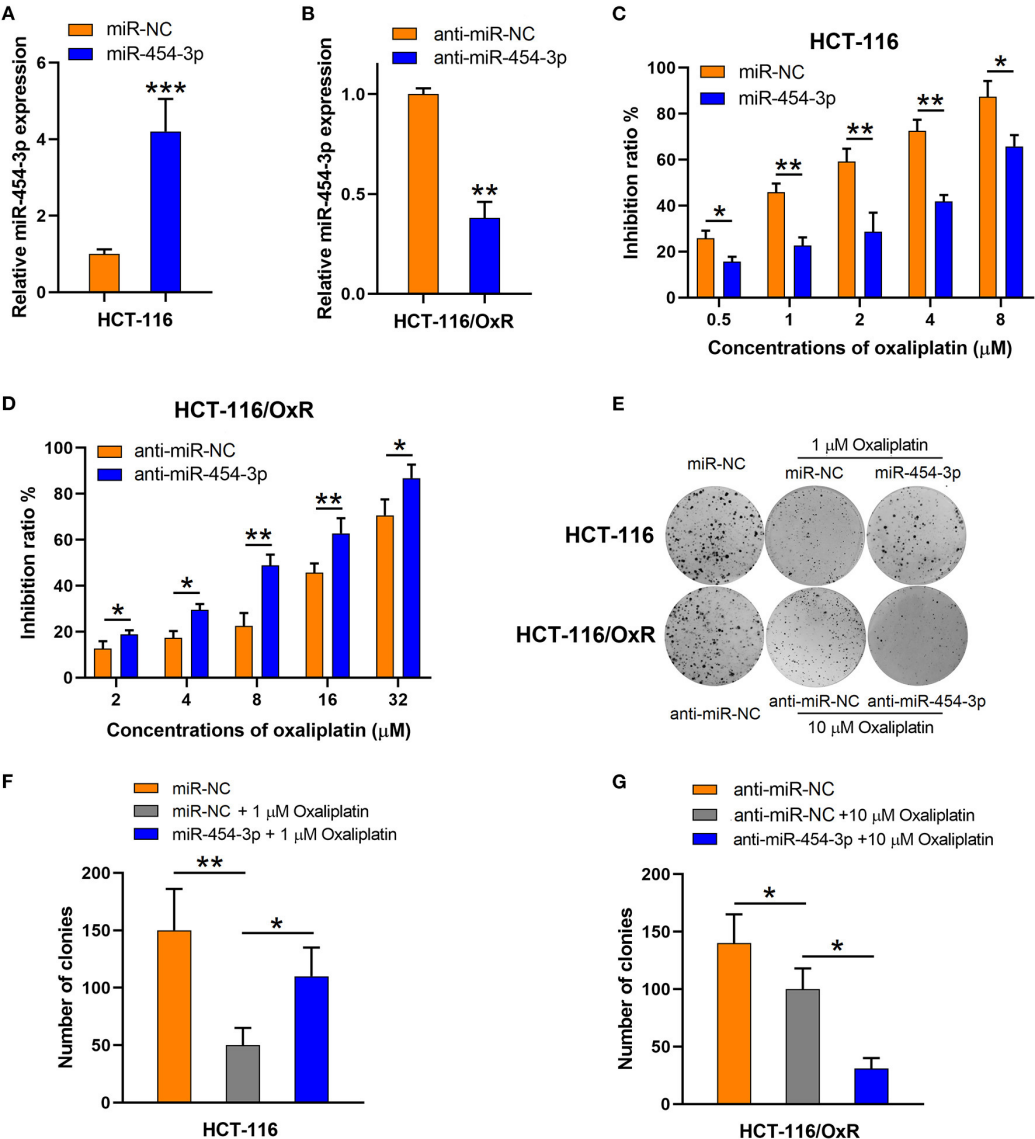
Altering miR-454-3p expression modulates cell sensitivity to oxaliplatin *in vitro*. **(A,B)** Quantitative RT-PCR analysis on relative miR-454-3p expression after miR-454-3p was overexpressed in HCT-116 cells **(A)** or knockdown in HCT-116/OxR cells **(B)**. **(C,D)** Inhibitory effects of oxaliplatin on cell viability determined by MTT assay. **(C)** HCT-116 cells transfected with miR-NC or miR-454-3p, and **(D)** HCT-116/OxR transfected with anti–miR-454-3p or anti–miR-NC. **(E–G)** Inhibitory effects of oxaliplatin on colony formation. **(E,F)** HCT-116 cells transfected with miR-NC or miR-454-3p, and **(E,G)** HCT-116/OxR transfected with anti–miR-454-3p or anti–miR-NC. Results were presented as mean ± SD from three independent experiments. *P < 0.05, **P < 0.01, ***P < 0.001.

To verify the influence of miR-454-3p on cellular sensitivity to oxaliplatin, we transfected miR-454-3p mimics or negative control miRNA (miR-NC) into HCT-116 cells (Figure 2A), whereas miR-454-3p inhibitor (anti–miR-454-3p) or inhibitor negative control (anti–miR-NC) was transfected into HCT-116/OxR cells (Figure 2B). Cell viability assay showed that overexpression of miR-454-3p significantly induced oxaliplatin resistance in HCT-116 cells, with the IC_50_ value increased from 1.14 ± 0.13 to 5.97 ± 1.14 μM (Figure 2C). In contrast, inhibition of miR-454-3p significantly sensitized HCT-116/OxR cells to oxaliplatin, with the IC_50_ value decreased from 21.22 ± 3.32 to 8.48 ± 1.37 μM (Figure 2D). In addition, a colony formation assay revealed that overexpression of miR-454-3p attenuated the oxaliplatin-mediated reduction of colonies in HCT-116 cells (Figures 2E,F), whereas the inhibition of miR-454-3p enhanced the inhibitory effects of oxaliplatin on colony formation in HCT-116/OxR cells (Figures 2E,G). Collectively, these results confirmed the positive correlation between the expression of miR-454-3p and oxaliplatin resistance in colorectal cancer cells.

### PTEN Was Identified as the Direct Target of miR-454-3p

**Figure 3:**
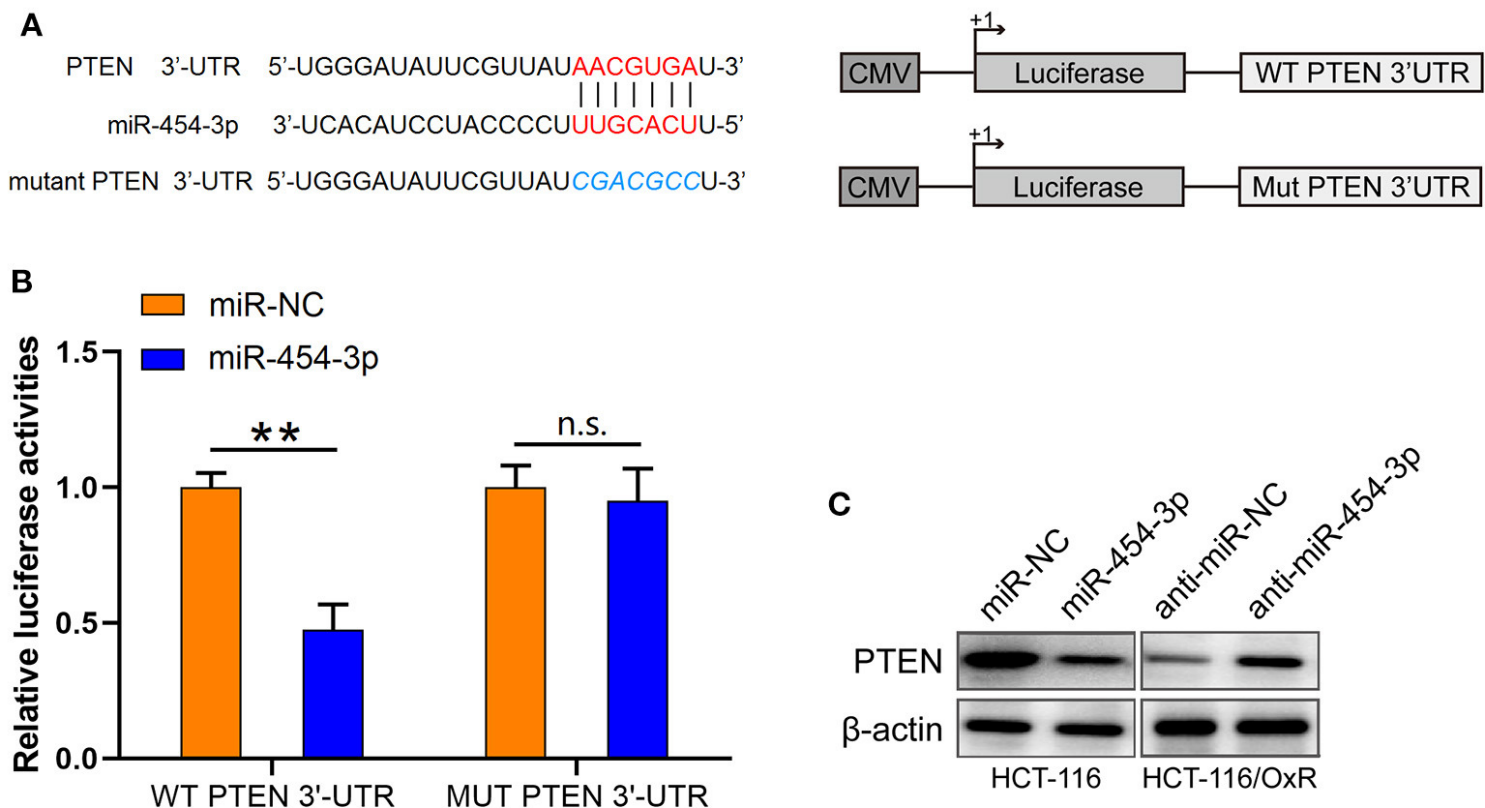
PTEN is a target of miR-454-3p. **(A)** Predicted miR-454-3p binding site in the 3′-UTR of PTEN. **(B)** Luciferase reporter assay confirmed that miR-454-3p bound to 3′-UTR of PTEN. **(C)** Western blotting assay showed the protein level of PTEN after overexpression or knockdown of miR-454-3p. **P < 0.01.

Based on the findings mentioned above, we further explored the molecular mechanisms underlying the effects of miR-454-3p on oxaliplatin sensitivity. Four online bioinformatics software programs (TargetScan, miRDB, miRCode, and doRiNA) were used to predict the target of miR-454-3p. A total of 12 common targeted mRNAs were found. Among these 12 targets, PTEN attracted our attention because it is a tumor suppressor, and its aberrant expression contributes to drug resistance and poor prognosis in several human cancers. Therefore, PTEN was chosen for further verification. The predicted correlation between the 3′-UTR of *PTEN* and miR-454-3p is shown in Figure 3A. To further confirm whether *PTEN* was the direct target of miR-454-3p, we used luciferase reporter assay to analyze the binding ability of miR-454-3p to wild-type or mutant 3′-UTR of PTEN. As shown in Figure 3B, overexpression of miR-454-3p significantly reduced the reporter activity of wild-type *PTEN* 3′-UTR, but the reporter activity of mutant *PTEN* 3′-UTR was not affected in HCT-116 cells, compared to that of miR-NC. Furthermore, a Western blot assay showed that overexpression of miR-454-3p mimics significantly down-regulated the expression of PTEN in HCT-116 cells, whereas the inhibition of miR-454-3p significantly up-regulated the expression of PTEN in HCT-116/OxR cells (Figure 3C). These results indicate that *PTEN* is the direct target of miR-454-3p.

### MiR-454-3p Inhibited Oxaliplatin-Induced Apoptosis by Down-Regulating PTEN Expression

**Figure 4:**
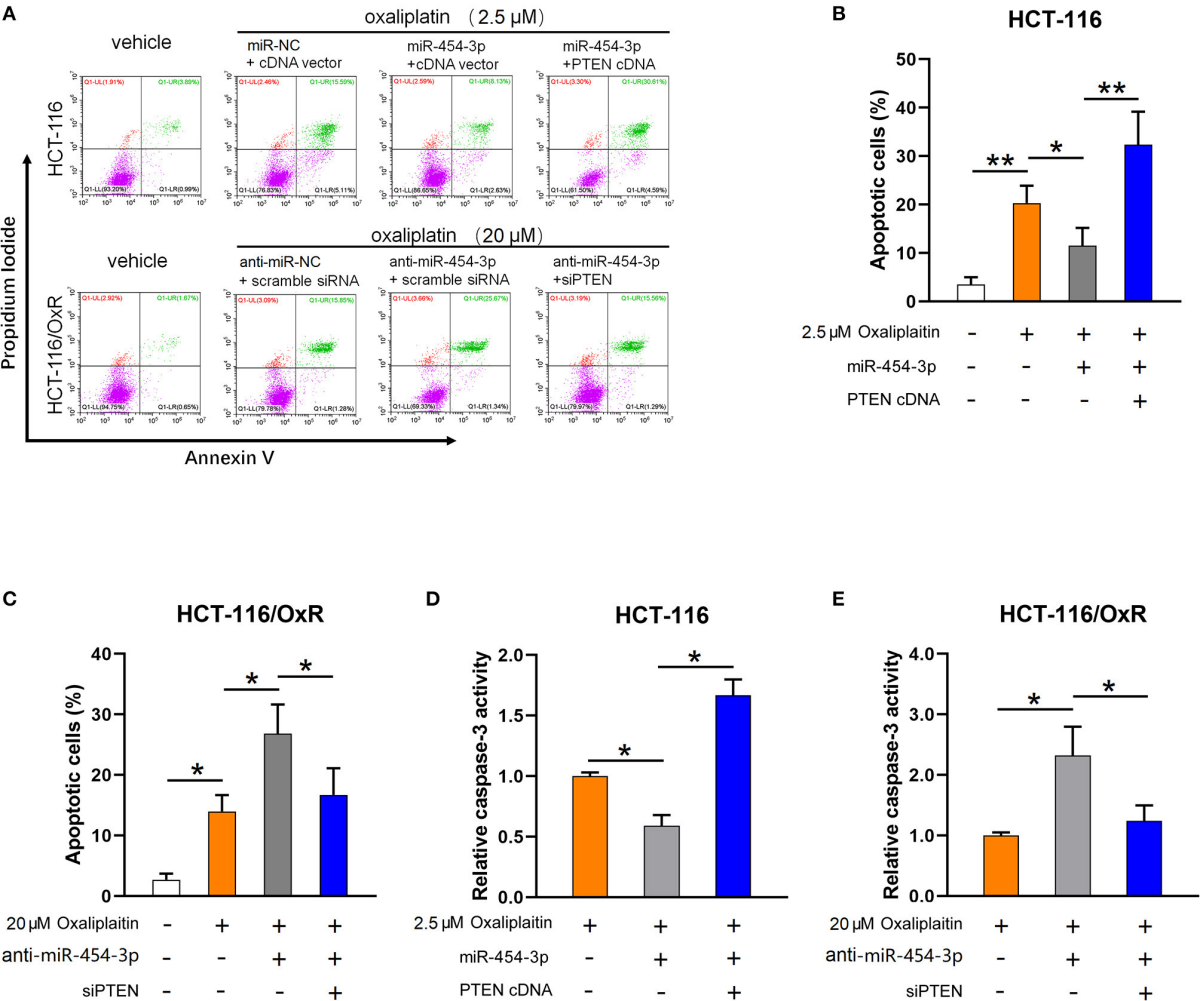
MiR-454-3p attenuates oxaliplatin-induced cell apoptosis through down-regulation of PTEN. **(A–C)** Cell apoptosis qualified by flow cytometry after annexin V–FITC and PI double staining in indicated cells treated with different concentrations of oxaliplatin. **(D,E)** Analysis of caspase-3 activity in indicated cells treated with different concentrations of oxaliplatin. Results were presented as mean ± SD from three independent experiments. *P < 0.05, **P < 0.01.

As the inhibition of apoptosis is considered as the main cause of cellular resistance to oxaliplatin (13, 14), we next sought to determine the effect of miR-454-3p on oxaliplatin-induced apoptosis in colorectal cancer cells. HCT-116 cells overexpressed miR-454-3p or miR-NC, and HCT-116/OxR cells transfected with anti–miR-454-3p inhibitor or anti–miR-NC were treated with 2.5 or 20 μM of oxaliplatin, followed by double-staining with annexin V–FITC and PI. Quantitative examination of cellular apoptosis by flow cytometry assay showed that overexpression of miR-454-3p inhibited oxaliplatin-induced cellular apoptosis, which was reversed by additional transfection of PTEN cDNA in HCT116 cells (Figures 4A,B). In contrast, inhibition of miR-454-3p increased the oxaliplatin-induced cellular apoptosis, which was reversed by further knockdown of PTEN in HCT-116/OxR cells (Figures 4A,C). Furthermore, we showed that overexpression of miR-454-3p significantly decreased caspase-3 activity, whereas the inhibition of miR-454-3p increased caspase-3 activity in HCT-116 or HCT-116/OxR cells, following treatment with oxaliplatin (Figures 4D,E). Moreover, overexpressing or silencing PTEN by the transfection of PTEN cDNA or siRNA partially reversed the effect of miR-454-3p on oxaliplatin-induced caspase-3 activity in HCT-116 or HCT-116/OxR cells (Figures 4D,E). Collectively, these results suggest that miR-454-3p limited oxaliplatin-induced apoptosis by down-regulating PTEN expression.

### MiR-454-3p Suppressed Apoptosis and Induced Oxaliplatin Resistance *via* Activation of AKT Pathway

**Figure 5:**
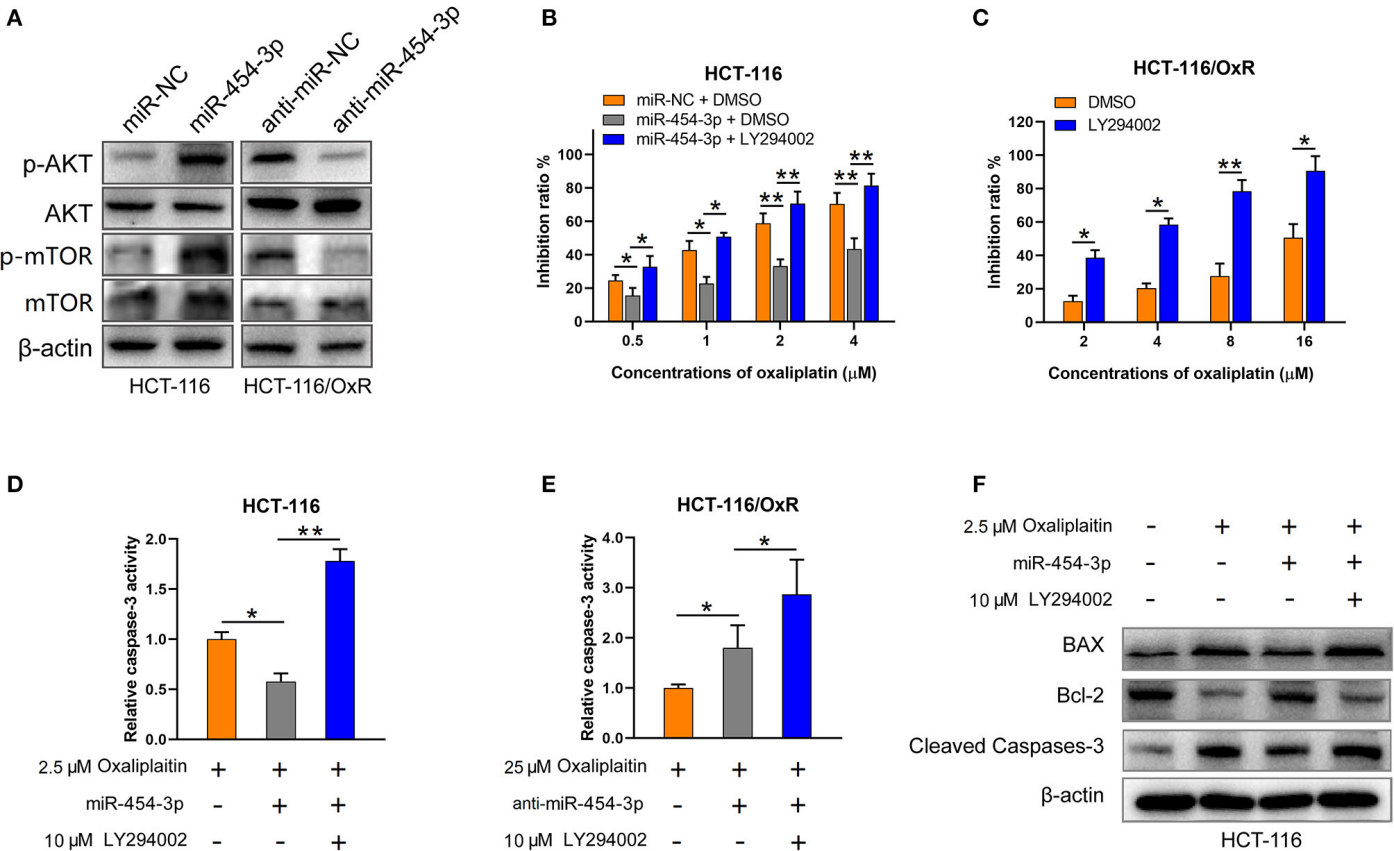
MiR-454-3p suppressed apoptosis and induced oxaliplatin resistance *via* activation of AKT pathway. **(A)** Western blotting on the expression of p-AKT, p-mTOR, total AKT, and total mTOR. β-Actin was used as an endogenous reference. **(B,C)** Cell viability determined by MTT assay in indicated cells treated with oxaliplatin and/or AKT signaling inhibitor LY294002. **(D,E)** Caspase-3 activity in indicated cells treated with different concentrations of oxaliplatin and/or LY294002. **(F)** Western blotting on the expression of apoptosis-related proteins, including Bax, Bcl-2, and cleaved caspase-3. Results were presented as mean ± SD from three independent experiments. *P < 0.05, **P < 0.01.

Since PTEN is a well-known suppressor of the PI3K/AKT/mTOR signaling pathway (15, 16), we next determined the effect of miR-454-3p on PI3K/AKT/mTOR pathway. As shown in Figure 5A, overexpression of miR-454-3p increased the phosphorylation of AKT and mTOR in HCT-116 cells, whereas its inhibition decreased the phosphorylation of AKT and mTOR in HCT-116/OxR cells. The expression of total AKT and mTOR was not affected by miR-454-3p. Furthermore, the PI3K inhibitor LY294002 was used to block the PI3K/AKT pathway activated by miR-454-3p. The result revealed that LY294002 reversed miR-454-3p–induced oxaliplatin resistance in HCT-116 cells (Figure 5B). Moreover, LY294002 resensitized HCT-116/OxR cells to oxaliplatin treatment (Figure 5C). In addition, LY294002 treatment also rescued the effect of miR-454-3p on oxaliplatin-induced caspase-3 activity in HCT-116 or HCT-116/OxR cells (Figures 5D,E). Apoptotic-related proteins Bcl-2, Bax, and cleaved caspase-3 were evaluated by Western blotting. As shown in Figure 5F, oxaliplatin treatment dramatically up-regulated the ratio of Bax/Bcl-2 expression and induced the cleavage of caspase-3, which were reversed by the overexpression of miR-454-3p. However, LY294002 treatment reduced the effects of miR-454-3p on the decrease of the Bax/Bcl-2 expression ratio and on the inhibition of cleavage of caspase-3. These results demonstrated that miR-454-3p suppresses oxaliplatin-induced apoptosis and leads to resistance to oxaliplatin *via* activating PTEN/AKT/mTOR pathway.

### MiR-454-3p Promoted Oxaliplatin Resistance in HCT-116 Colorectal Cancer Cells *in vivo*

**Figure 6:**
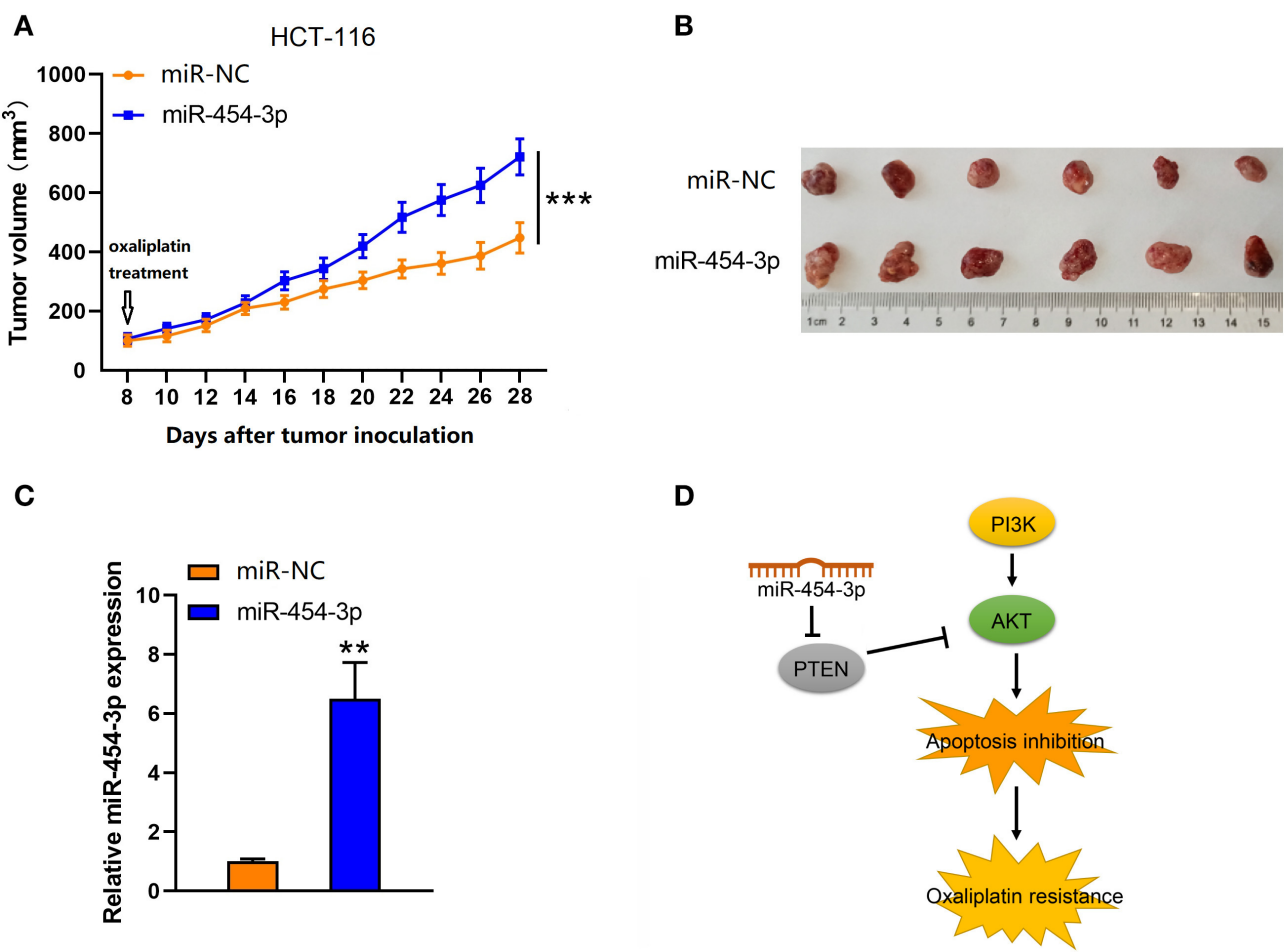
Up-regulation of miR-454-3p reduced the sensitivity of colorectal cancer cells to oxaliplatin *in vivo*. **(A)** Tumor xenografts with HCT-116 cells stably transfected with miR-454-3p or negative control. The mice received oxaliplatin every 4 days starting from the 8th day after tumor inoculation. **(B)** Representative images of dissected tumors in each group. **(C)** Quantitative RT-PCR analysis of the expression of miR-454-3p in representative tumor tissues. **(D)** A proposed mechanism of miR-454-3p mediated oxaliplatin resistance. **P < 0.01, ***P < 0.001.

To further investigate the effect of miR-454-3p on oxaliplatin resistance *in vivo*, colorectal cancer xenograft models were established by subcutaneously injecting mice with HCT-116 cells that were stably overexpressed miR-454-3p or negative control miRNA (miR-NC). After the tumor value reached 100 mm^3^ (8 days after tumor inoculation), the mice began receiving oxaliplatin by intraperitoneal injection every 4 days. As shown in Figures 6A,B, the miR-NC–transfected tumors grew slower than the miR-454-3p–overexpressed tumors, indicating that miR-454-3p reduced the sensitivity of colorectal cancer cells to oxaliplatin treatment *in vivo*. Quantitative RT-PCR analysis confirmed the expression of miR-454-3p in HCT-116 cells from representative tumor tissues (Figure 6C). These *in vitro* and *in vivo* results revealed that miR-454-3p inhibited oxaliplatin induced-apoptosis and promoted oxaliplatin resistance in colorectal cancer by repressing PTEN expression, thereby enhancing PI3K/AKT signaling pathway (Figure 6D).

### MiR-454-3p Expression Negatively Correlates With PFS in Colorectal Cancer Patients Who Received Oxaliplatin-Based Chemotherapy

**Figure 7:**
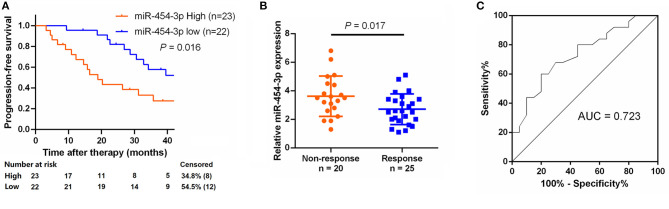
Expression of miR-454-3p correlates with PFS in oxaliplatin treated colorectal cancer patients. **(A)** Colorectal cancer patients who received oxaliplatin-based chemotherapy were separated into groups based on low or high miR-454-3p expression. Kaplan–Meier survival curves were used to compare the PFS between the two groups. **(B)** Expression of miR-454-3p in patients who responded to oxaliplatin (*n* = 25) and who did not respond (*n* = 20) to oxaliplatin was compared using the two-tailed Student *t* test. *U6* small nuclear RNA was used as an internal control. **(C)** ROC curve for patients responding vs. non-responding to oxaliplatin treatment. The AUC value was 0.723, yielding sensitivity of 71.0% and specificity of 73.2% at the cutoff value of 3.1.

To study the relationship between miR-454-3p and oxaliplatin resistance in colorectal cancer, we selected 45 patients who had received oxaliplatin-based chemotherapy and stratified these individuals into **two** groups of high and low miR-454-3p expression, according to the expression of miR-454-3p in their tumor tissues by quantitative RT-PCR (Table 1). Kaplan–Meier analysis showed that colorectal cancer patients with high miR-454-3p expression exhibited shorter PFS than those with low miR-454-3p expression (Figure 7A). Moreover, miR-454-3p expression was significantly more up-regulated in patients with non-response to chemotherapy than in those who showed a response (Figure 7B). The receiver operating characteristic curve was conducted to assess the predictive value of miR-454-3p expression for response to oxaliplatin. As shown in Figure 7C, the area under the curve (AUC) value was 0.723, yielding sensitivity of 71.0% and specificity of 73.2% at the cutoff value of 3.1 (Figure 7C). These data suggest the important role of miR-454-3p in promoting oxaliplatin resistance in colorectal cancer.

## Discussion

Oxaliplatin resistance has been recognized as one of the major obstacles preventing the effective therapy in colorectal cancer; however, the underlying molecular mechanism remains largely unclear. A growing number of studies have indicated that aberrant miRNA expression modulates genes related to chemosensitivity or chemoresistance in various human malignancies, including colorectal cancer (17–21). A previous study showed that natural killer cells inhibit oxaliplatin-resistant colorectal cancer by repressing WBSCR22 *via* up-regulating miR-146b-5p (22). In another study, exosome-mediated miR-128-3p delivery was demonstrated to inhibit oxaliplatin-induced epithelial–mesenchymal transition (EMT) and enhanced oxaliplatin response through negative regulation of Bmi1 and MRP5 in colorectal cancer cells (23). In the present study, we compared the expression of a panel of miRNAs between oxaliplatin-sensitive and -resistant HCT-116 colorectal cancer cells by quantitative RT-PCR. Fifteen miRNAs were identified to have more than 2-fold difference in expression between oxaliplatin-sensitive and -resistant cells, including several miRNAs that have been previously reported to be associated with oxaliplatin sensitivity in colorectal and other cancer cells. For instance, it has been reported that miR-153 (24) and miR-20a (25) enhanced oxaliplatin resistance by inhibiting FOXO3a and BNIP2 in colorectal cancer, whereas miR-122 (26, 27) sensitizes hepatocellular carcinoma to oxaliplatin by inhibiting the Wnt/β-catenin pathway.

In this study, we observed that miR-454-3p was the most differently expressed miRNA and significantly up-regulated in oxaliplatin-resistant cells. Furthermore, we showed that the inhibition of miR-454-3p resensitized oxaliplatin-resistant cells to oxaliplatin by enhancing its inhibitory effect on cell viability and colony formation, suggesting the essential role of miR-454-3p in regulating oxaliplatin resistance. Several studies have partially described the mechanisms of miR-454-3p in the promotion or suppression of cancer progression. It has been reported that miR-454-3p promotes breast cancer metastasis through targeting nuclear precursor mRNA domain-containing 1A (RPRD1A), a known tumor suppressor, thereby activating Wnt/β-catenin signaling pathway (28). In cervical cancer, miR-454-3p was found to promote cell proliferation and inhibit apoptosis by targeting tripartite motif-containing 3 (TRIM3) (29). Conversely, miR-454-3p was also observed to act as a tumor suppressor in bladder cancer (30) and non–small cell lung cancer (31) by targeting the EMT inducer ZEB2 or constituent Ca^2+^-binding protein calbindin 1 (CALB1), respectively. However, the association between miR-454-3p and oxaliplatin resistance is unknown. Therefore, we chose miR-454-3p to further assess its function and directly target the relevant genes in oxaliplatin resistance.

Using a bioinformatics method, together with experimental approaches, we identified *PTEN* as the direct target of miR-454-3p, which was further confirmed by luciferase reporter assay and Western blot analysis. PTEN is a well-characterized tumor suppressor that acts by negatively regulating the PI3K/AKT signaling pathway. Increasing evidence has demonstrated that down-regulation or deficiency of PTEN, resulting in hyperactivation of the PI3K/AKT signaling pathway, is closely associated with resistance to platinum-based chemotherapy, including oxaliplatin (32–34). Our study showed that overexpression of miR-454-3p down-regulated PTEN expression, but promoted phosphorylation of AKT and mTOR, leading to a reduction in oxaliplatin sensitivity. In contrast, inhibition of miR-454-3p up-regulated PTEN expression, but attenuated the phosphorylation of AKT and mTOR, resulting in resensitization to oxaliplatin. Notably, previous studies reported that miR-454-3p inhibited AKT signaling pathway by targeting insulinlike growth factor 2 mRNA-binding protein 1 (IGF2BP1) in esophageal cancer (ESCA) (35) and targeting c-met in nasopharyngeal carcinoma (36), resulting in the suppression of cell growth and enhancement of cisplatin sensitivity. These inconsistent results may indicate that the role of miR-454-3p in modulating tumor progression and chemotherapeutic sensitivity was dependent on the types of human malignancies.

Several studies have indicated that, once phosphorylated by PI3K, activated AKT has direct effects on the regulators of the apoptotic pathway, including Bcl-2 family members, as such activation of PI3K/AKT signaling could up-regulate Bcl-2 expression and enhance its activity, leading to increased cell survival and reduced chemotherapy-induced apoptosis (37–39). Apoptosis is a major form of cell death, which is tightly regulated by the interaction between antiapoptotic and proapoptotic proteins, particularly the Bcl-2 family (40). The ratio between antiapoptotic and proapoptotic Bcl-2 family proteins has been recognized as an effective indicator to judge whether a cell will progress to apoptosis (41). In the current study, we demonstrated that the overexpression of PTEN or the use of the specific AKT signaling inhibitor LY294002 could overcome miR-454-3p–mediated oxaliplatin resistance and further enhance oxaliplatin-induced cellular apoptosis by decreasing the expression ratio of antiapoptotic factor Bcl-2 to proapoptotic factor Bax.

It has also been suggested that miR-454-3p may be used as a prognostic biomarker in different types of cancers. Ren et al. (28) reported that miR-454-3p could be an independent prognostic factor in breast cancer, and patients with higher miR-454-3p expression experienced poorer overall survival and shorter PFS times. Meanwhile, Li et al. (42) found that overexpression of miR-454-3p was significantly correlated with poor prognosis in hepatocellular carcinoma patients. In contrast, Shao et al. (43) observed that overexpression of miR-454-3p in exosomes in plasma and low miR-454-3p expression in tumor tissue were associated with poor prognosis, indicating the functions of miR-454-3p as a tumor suppressor in glioma. Our present study indicated that overexpression of miR-454-3p suggested short PFS in colorectal cancer patients who received oxaliplatin-based chemotherapy, supporting the potential application of miR-454-3p to predict the prognosis of oxaliplatin-based chemotherapy. After being further validated, the measurement of miR-454-3p in colorectal tumor tissues would be used for the prediction of oxaliplatin treatment response and for the selections of chemotherapeutic regimen. In addition, silencing miR-454-3p by chemical compounds could be a novel therapeutic strategy to sensitize colorectal tumor cells to oxaliplatin treatment.

Although several miRNAs were previously reported to be involved in oxaliplatin resistance in colorectal cancer cells through multiple molecular pathway (9–12), including PTEN-AKT signaling pathway (32–34), to our knowledge, there is no investigation implicating miR-454-3p in chemoresistance of colorectal cancer. Our current study identified miR-454-3p as a critical factor of oxaliplatin resistance in colorectal cancer cells by comparing its expression between oxaliplatin-sensitive and -resistant colorectal cancer cells. In particular, we proved that miR-454-3p directly binds to 3′-UTR of *PTEN* and reduces its expression by analyzing the binding ability of miR-454-3p to wild-type or mutant *PTEN* 3′-UTR.

In conclusion, our study provides evidence that miR-454-3p promoted oxaliplatin resistance in colorectal cancer cells by targeting PTEN expression, which negatively regulated PI3K/AKT signaling pathway. Inhibition of miR-454-3p or restoration of PTEN expression resensitized colorectal cancer cells to oxaliplatin through attenuating PI3K/AKT signaling pathway. Clinically, overexpression of miR-454-3p was associated with a decreased responsiveness to oxaliplatin-based chemotherapy, as well as a short PFS. These data indicated that miR-454-3p possesses the potential of predicting oxaliplatin sensitivity and could be a novel therapeutic target to overcome oxaliplatin resistance in colorectal cancer.

## Data Availability Statement

The original contributions presented in the study are included in the article/supplementary material, further inquiries can be directed to the corresponding author/s.

## Ethics Statement

The studies involving human participants were reviewed and approved by Medical Ethics Committee of Zhangjiagang First People's Hospital. The patients/participants provided their written informed consent to participate in this study. The animal study was reviewed and approved by Medical Ethics Committee of Zhangjiagang First People's Hospital.

## Author Contributions

X-LQ, YZ, and CN designed the projects and wrote the manuscript. X-LQ, FZ, SX, JJ, Z-PC, and S-KW performed the experiments and analyzed the results. All authors read and agreed the final manuscript.

## Conflict of Interest

The authors declare that the research was conducted in the absence of any commercial or financial relationships that could be construed as a potential conflict of interest.
